# A New Measure for Assessing Substance-Related and Addictive Disorders: The Addictive Behavior Questionnaire (ABQ)

**DOI:** 10.3390/jcm7080194

**Published:** 2018-08-01

**Authors:** Vincenzo Caretti, Alessio Gori, Giuseppe Craparo, Marco Giannini, Giuseppe Iraci-Sareri, Adriano Schimmenti

**Affiliations:** 1 Department of Human Sciences—LUMSA University of Rome Borgo Sant’Angelo, 13, 00193 Rome—Italy and Institute of Integrative Psychodynamic Psychotherapy, Via Ricasoli 32, 50122 Florence, Italy; vincenzocaretti@gmail.com; 2 Faculty of Human and Social Sciences, UKE—Kore University of Enna, Cittadella Universitaria, 94100 Enna, Italy; giuseppe.craparo@unikore.it (G.C.); adriano.schimmenti@unikore.it (A.S.); 3 Department of Health Sciences, University of Florence, 50135 Florence, Italy; giannini@psico.unifi.it; 4 Institute of Integrative Psychodynamic Psychotherapy, Italian Society of Clinical Psychodiagnosis, 50135 Florence, Italy—Gruppo Incontro, 51100 Pistoia, Italy; giuseppe.iraci@incontro.coop

**Keywords:** addiction, addictive behaviors, assessment, treatment

## Abstract

This article evaluates the psychometric properties of a new measure for assessing Substance-Related and Addictive Disorders: the Addictive Behavior Questionnaire (ABQ). The ABQ is a self-report measure composed of two sections: the Severity Index (SI) and the Seven Domains Addiction Scale (7DAS). Materials and methods. A total sample of 698 subjects divided into two groups (515 subjects in the clinical sample and 183 subjects in the control sample), participated in this study. We applied Exploratory Factor Analysis (EFA) to examine features of ABQ construct validity, we used Cronbach’s alpha coefficient to assess its internal reliability, and explored some aspects of its concurrent validity by examining its associations with other measures assessing addictive behaviors and psychopathology. Results and conclusions: results of EFA indicated that all the scales of the ABQ are unidimensional and showed good internal consistency. The correlations between the sections of the ABQ and the other measures used in the current study were significant and in the expected directions. These results suggest that the ABQ has good psychometric properties and allows researchers and clinicians to gather relevant information regarding behaviors, psychopathology and severity of symptoms, for the best clinical reasoning and for planning tailored treatment for each patient.

## 1. Introduction

In the fifth edition of the Diagnostic and Statistical Manual of Mental Disorders (DSM-5) [1], a section devoted to substance-related and addictive disorders was introduced, in which both substance use and gambling disorders are included. This novel categorization of these disorders into a single diagnostic class indicates that the classic construct of addiction no longer refers only to problems related to the recursive and uncontrollable use of a particular substance. This terminological revision testifies the sensitivity of the DSM-5 Task Force toward the clinical and research observations showing that addictive tendencies may develop in relation to both substances and behaviors. According to these observations, gambling disorder was included in this diagnostic class of substance-related disorders and addictive disorders, and Internet gaming disorder was introduced as a condition for further study. Similarly, terms such as abuse and dependence, deeply rooted in the clinical tradition, converge today in this single picture of substance-related disorders and addictive disorders, to describe the wide range of the disorder, from mild forms to severe states with chronic relapses. However, the DSM-5 maintains its attention to the current severity of addictive disorders via its specifiers (i.e., it allows the assessment of mild, moderate, or severe degree of the disorder). Moreover, the current attention to the craving phenomena in the DSM-5 is in line with clinical wisdom suggesting that this feature is as much meaningful as the tolerance and withdrawal symptoms for both diagnostic evaluation and treatment planning of addictive behaviors. Tolerance can be defined as a need for increased amounts of the substance or behavior to achieve the desired effect. This feature is commonly observed in addicted individuals; however, tolerance varies greatly from individual to individual, for example, due to the different physical characteristics that can make one subject more sensitive than another to a substance [2]. A similar reasoning applies to withdrawal symptoms (i.e., those symptoms occurring upon the abrupt discontinuation or decrease in intake of substances or in enacting the behavior), as they can greatly vary in relation to the specific substances and behaviors. Moreover, symptoms of tolerance or withdrawal may also occur during appropriate treatments with prescribed drugs (for example, analgesic opioids, sedatives, stimulants), which poses serious questions about specifically relying on these symptoms for a diagnosis of substance use disorder.

From a more phenomenological perspective, the domain of impulsivity, obsessiveness, and compulsivity may have a similar relevance with respect to tolerance and withdrawal for the understanding of addictive behaviors. In fact, many people displaying problematic substance use and/or excessive behaviors may be obsessed with thoughts, memories, and desires concerning their addictive behaviors (obsessiveness), and they might impulsively enact them for achieving pleasure (impulsivity) and/or they might feel forced to enact them to avoid unpleasant feelings (compulsivity). Paradoxically, addictive behaviors may provide some stability to the needs of many addicted individuals, as they allow these individuals to obtain an immediate gratification of dysregulated urges and impulses on one side, and to tolerate otherwise painful or unbearable affect states on the other side [3]. According to this perspective, addictive behaviors may represent a mental retreat for the addicted individual that provide a psychological way to escape from the difficulty and the unpredictability of human life, a sort of mental anesthetic that allows the individual to counteract physical and psychological dysregulation, albeit dysfunctionally [4].

The developmental environment of an individual plays a critical role in the development of this vulnerability to addictive behaviors. In fact, a positive environment and supportive relationships in childhood and adolescence may represent a protective factor with respect to the onset of substance use or addictive-related disorders: as the studies on resilience indicate [5], relational contexts characterized by affection, positive communication and prosociality reduce the probability of developing risky behaviors and psychological disorders. On the contrary, when the developmental environment is pathological in itself, e.g., when it is characterized by phenomena such as neglect, abuse, violence and widespread anti-social behavior, it multiplies the vulnerability to addictive behaviors. Thus, traumatic experiences in the attachment relationships during childhood, and the insecure attachment styles that stem from such experiences, may constitute further risk factors for the development of addictive behaviors [6,7,8]. This is because the emotional dysregulation resulting from such negative experiences may negatively affect the development of, and interactions among brain structures that are relevant for understanding oneself and one’s own actions, including the hippocampal region involved in the consolidation of memory and the prefrontal cortex involved in executive functioning and decision making, among many other structures damaged by childhood trauma [9]. Such vulnerability to emotional dysregulation may also become embedded in the individual personality and way of relating with others [10], thus fostering both severe difficulties in processing and integrating distressing experiences at the mental and bodily level, and severe distrust of others for receiving protective closeness and interpersonal regulation [11].

Such condition of emotional, cognitive, and relational dysregulation might in turn induce the individual to the obsessive, impulsive and compulsive use of a substance or a behavior, which allows a defensive withdrawal into mental states that are dissociated (i.e., not integrated) from the ordinary consciousness. The withdrawal into these states by means of substance and/or behaviors may temporarily allow the addicted individual to push away the dysregulated emotions and traumatized mental states from the awareness. However, the escape into the temporary retreat of substance use and/or excessive behaviors further weakens the capacity for self- regulation of affects and fuels instead the impulsive need to reenact the addictive behavior to achieve again the sensation of pleasure and the reduction in the intensity of negative affect states. This creates a vicious circle, in which the reinforcing memory of the pleasure for the behavior and the compulsive ritualization of the behavior aimed to reducing pain, feed the thoughts and the obsessive urge to repeat the behavior, despite the negative effects that such action may produce on the individual’s psychological and physical health. Some studies have partially confirmed these clinical speculations [12,13,14,15,16].

According to this theoretical model of addiction, we consider addictive behaviors as disorders resulting from the interactions between impulsivity, compulsivity, obsessiveness, emotional dysregulation, traumatized mental states, dissociation, and insecure attachment styles. Therefore, these domains of psychological functioning may represent key features to make a careful assessment and an individualized treatment plan for people suffering from addictive behaviors.

In line with this new model of addiction, it was important to develop a measure for assessing addictions that can be used in professional practice with clinical populations and as a screening tool for nonclinical populations. Besides, in our context, there is a lack of self-report measures that assess addictive behaviors in a complex, multidimensional manner in both clinical and nonclinical populations. Thus, in the development of this new measure we paid attention to several criteria:

(1) Categorical assessment of addictive behaviors. The ABQ evaluates the presence of substance-related disorder, gambling disorder, and problematic Internet use.
(2) Dimensional assessment of addictive behaviors in relation to their severity level. The ABQ allows clinicians to have a specific dimensional picture of the severity of the addictive behaviors, to improve the professional reasoning and the decision making.
(3) Assessment of psychopathological and behavioral variables. The ABQ measures seven domains related to addictive behaviors, according to the theoretical model behind the measures that conceives addictive behaviors as the results of an interaction between impulsivity, compulsivity, obsessiveness, emotional dysregulation, traumatized mental states, dissociation, and insecure attachment styles.
(4) Good psychometric properties.

In this paper, we present the Addictive Behavior Questionnaire (ABQ) and examine the main psychometric properties of the ABQ in an Italian sample.

## 2. Materials and Methods

### 2.1. Participants and Procedure

A total sample of 698 subjects (209 females, 482 males, missing values 7), with a mean age of 36.44 years old (SD = 11.77) participated in this study. Participants were divided into two groups: a clinical sample composed of 515 subjects (111 females, 398 males, missing values 6—mean age 39.03, SD = 11.64) and a control sample of 183 subjects (98 females, 84 males, missing values 1—mean age 29.09, SD = 8.62) (see Table 1). The clinical sample was mainly recruited at the National Health System (NHS) in various Italian Regions with the collaboration of the various National Health Drugs Services (Ser.D. Italy) and FeDerSerD (Italian Federation of Dependency Departments and Services Operators). Significant differences were found between groups concerning age (*χ*^2^ = 162.50, *p* < 0.001) gender (*χ*^2^ = 65.23, *p* < 0.001) and years of education (*χ*^2^ = 150.17, *p* < 0.001). The recruitment of patients was made possible thanks to the collaboration of various organizations (social cooperative) that work in the field of addictions. Each participant of the clinical group received a clinical diagnosis based on the DSM-5 that falls in the diagnostic class of “Substance-Related and Addictive Disorders”. In addition to ABQ, participants completed other questionnaires described in the measure section. The questionnaires were administered according to the laws of privacy and informed consent of the Italian law (Law Decree DL-196/2003) by the professionals of the services. The participants were also told that they could withdraw from the study at any time and that there would be no payment for participating in the study. Regarding ethical standards for research, the study followed procedures consistent with the latest version of the Declaration of Helsinki revised in Fortaleza [17].

### 2.2. Measures

Demographics questionnaire. A brief demographic survey was administered, assessing information concerning age, gender, area of residence, relationship status, education, employment status and ethnicity.

Addictive Behavior Questionnaire (ABQ) [2]. The ABQ is a self-report measure that was designed as a support for the assessment of the different forms of addiction in all phases of the intervention. The ABQ consists of two instruments: The Severity Index (SI) and the Seven Domains Addiction Scale (7DAS). The two instruments are preceded by a personal data sheet and a section to identify the presence and frequency of both substance use (such as alcohol, opioid, etc.) and excessive behaviors (pathological gambling and problematic Internet use). The SI is used to assess the current severity of addiction in four main areas: (1) psychoactive substances; (2) alcohol; (3) gambling; (4) Internet. Each area is composed of two sections: the section A for the assessment of the addictive behavior; the section B for the assessment of seven domains of psychopathology that are relevant for the understanding and treatment of addictive behaviors. The Seven Domains Addiction Scale (7DAS) included in this section B measures seven different features of mental and behavioral functioning that have been considered according to clinical experience and research findings as risk factors for the development and maintenance of addictive behaviors. Each domain consists of 7 questions with a 5-point Likert scale: Domain 1, Separation anxiety, for the evaluation of difficulties to safely create and/or enjoy an attachment relationships (insecure attachment); Domain 2, Affect dysregulation, for the evaluation of the difficulty to identify, differentiate, modulate, and communicate emotions; Domain 3, Somatoform and psychological dissociation, for the evaluation of symptoms of altered state of consciousness and strangeness toward oneself, one’s own body and others; Domain 4, Childhood traumatic experiences, for the evaluation of traumatized mental states related to experiences of emotional neglect or abuse during childhood; Domain 5, Impulse Dyscontrol, for the assessment of the difficulty to resist an impulse, an impelling desire or behavior, despite the potential negative consequences of one’s actions; Domain 6, Compulsive behavior and ritualization, for the evaluation of the tendency to compulsively implement repetitive and ritualized behaviors, even when they are dangerous for one’s own health or well-being; Domain 7, Obsessive thoughts, for the evaluation of obsessional, recurrent, persistent and repetitive thinking and difficulty to make a decision.

South Oaks Gambling Screen (SOGS) [18]. The SOGS is a psychometric instrument widely used internationally to assess the presence and severity of pathological gambling (PG). Respondents scoring 3 and 4 are classified as “problem gamblers”, and those scoring 5 points or more are classified as “pathological gamblers”. The SOGS was found to have satisfactory reliability with coefficient alphas of 0.69 and 0.86 in the general population and gambling treatment samples, respectively [19]. The SOGS demonstrated also satisfactory validity, by differentiating between the general population and the gambling treatment sample and by exhibiting high correlations with DSM-IV diagnostic criteria and moderate correlations with other measures of gambling problem severity [19]. In the present study we used the Italian version of Guerreschi and Gander [20].

Barratt Impulsiveness Scale (BIS-11) [21]. The Barratt Impulsiveness Scale-11 (BIS-11) is a 30 item self-report questionnaire designed to assess general impulsiveness taking into account the multi-factorial nature of the construct. The structure of the instrument allows for the assessment of six first-order factors (attention, motor, self-control, cognitive complexity, perseverance, cognitive instability) and three second-order factors: attentional impulsiveness (attention and cognitive instability), motor impulsiveness (motor and perseverance), non-planning impulsiveness (self-control and cognitive complexity). A total score is obtained by summing the first or second-order factors. The items are scored on a four-point scale (Rarely/Never = 1, Occasionally = 2, Often = 3, Almost Always/Always = 4). In the present investigation we used the Italian version of Fossati and colleagues [22].

Twenty-Items Toronto Alexithymia Scale (TAS-20) [23,24,25]. The TAS-20 consists of twenty items which load on three factors. These three factors are denoted as F1 “Difficulty in identifying feelings,” F2 “Difficulty in describing feelings,” and F3 “Externally-oriented thinking.” Fifteen items are indicative of the dimensions of alexithymia and five are contra-indicative. The rating scales have five response categories varying from “strongly disagree” (1) to “strongly agree” (5). A total score is calculated by summing all items such that higher score reflects a greater level of alexithymia. Scores higher than 61 are categorized as indicating an alexithymic profile according to the recommendation of Taylor et al. [24]. The original TAS-20 is characterized by acceptable psychometric qualities. The reliability of the total scale equals 0.81, and the reliabilities of the three factors are 0.78, 0.75, and 0.66 (F1, F2, F3 respectively) [23,24]. The validity of the TAS-20 is also acceptable [23,24]. In this study, we used the Italian version of the TAS-20 [26].

Dissociative Experience Scale-II (DES-II) [27]. Dissociative symptoms were assessed using the 28-item self-report Dissociative Experiences Scale-II (DES-II). This scale reflects severity of psychological dissociation, addressing the frequency of experiences of amnesia (e.g., gaps in memory), absorption (level of focus on internal or external cues), and depersonalization/derealization (impairments in sensing that the self or the world is real). Responders are asked to rate various dissociative experiences that are occurring in their daily life. Each item is rated on a 0–100% scale and the individual’s score is the mean score of the 28 items. Higher scores indicate greater levels of psychological dissociation. Carlson and Putnam [27] concluded that the scale has good psychometric properties. In the present study we used the Italian version of the DES-II [28].

Somatoform Dissociation Questionnaire (SDQ-5) [29]. The SDQ-5 is a 5-item dissociative disorders screening instrument (range of scores 5–25), and was introduced as above. The recommended cutoff point is ≥8. The SDQ-5 provided optimal discrimination between cases of dissociative disorders and psychiatric patients with other DSM-IV disorders, and yielded high sensitivity (94%) and specificity (96%) [29].

Psychological Treatment Inventory—Attachment Styles Scale [30]. The Psychological Treatment Inventory Attachment Styles Scale (PTI-ASS), is a section of the Psychological Treatment Inventory [31] that was designed to measure the quality of attachment relationship people invest as well as the correlated behaviors, emotions and thoughts, which could be derived from conscious drives. By evaluating these components, the PTI-ASS assesses the related attachment style from among the categories of secure, preoccupied, avoidant and disorganized. The intent is to infer the linked attachment style via assessing conscious derives [30]. The PTI-ASS is composed of 22 items, with a Likert scale with five points (from 1 = “Not at All” to 5 = “A Great Deal”), that are intended to assess the related attachment style from among the categories of Secure, Preoccupied, Avoidant and Disorganized. The factor structure, reliability, construct, and concurrent validity of the PTI-ASS have been verified in a previous study [30].

Internet Addiction Test (IAT) [32]. The IAT is one of the most common diagnostic instruments for Internet addiction. It comprises 20 items rated in a five-point Likert scale (from 1—not at all, to 5—always). This instrument is derived from the DSM-IV criteria for PG and alcoholism and it measures the extent of individual’s problems due to the Internet use in daily routine, social life, productivity, sleeping patterns, and feelings. Based on the total score obtained on the test; participants may fall into the categories of average online users who has a full control of his or her usage (below 50); excessive Internet users (between 50 and 79), people suffering from Internet addiction (80 or above) [33].

Yale-Brown Obsessive Compulsive Scale (Y-BOCS) [34,35]. The Y-BOCS is self-rating scale is designed to assess the severity and type of symptoms in patients with OCD. The Y-BOCS comprises a Symptom Checklist and Severity Scale to consecutively rate obsessions and compulsions. The Symptom Checklist includes common obsessions and compulsive behaviors, which are grouped according to thematic content. Symptoms that are rated using a five-point scale ranging from 0 (none) to 4 (extreme) across five dimensions: (1) time/frequency, (2) interference, (3) distress, (4) resistance, and (5) degree of control. Obsessive and compulsive symptom severity are rated separately (scores range from 0 to 25) with these scores summed to create a total OCD severity score (range, 0–50). The following score clusters approximately map onto symptom severity: mild symptoms (0–13), moderate symptoms (14–25), moderate–severe symptoms (26–34), and severe symptoms (35–40) [36].

Alcohol Use Disorders Identification Test (AUDIT) [37]. The AUDIT is a 10-item screening tool developed by the World Health Organization (WHO) to assess alcohol consumption, drinking behaviors, and alcohol-related problems. AUDIT provides a simple method of early detection of hazardous and harmful alcohol use. The AUDIT’s 10 multiple-choice response items require approximately 2 min for administration; a total score of eight or more indicates a strong likelihood of harmful alcohol consumption. The AUDIT has been shown to be a valid instrument for identifying alcohol disorders.

Traumatic Experiences Checklist (TEC) [38]. The TEC is a self-report measure that assesses 29 potentially traumatic events, such as emotional abuse, neglect, sexual assault, and physical abuse. The TEC also addresses trauma severity across the following four variables: (1) event occurrence; (2) early traumatic experiences; (3) the duration of trauma; (4) subjective reaction to trauma (4). In this study, we used the Italian version of the TEC by Schimmenti (2017) [39].

### 2.3. Data Analysis

Descriptive statistics for clinical and control samples were calculated. Besides, Exploratory Factor Analysis (EFA) was applied. We first analyzed the Bartlett’s Test of Sphericity and the Kaiser-Meyer-Olkin’s (KMO) Measure of Sampling Adequacy to assess if the items were significantly correlated and shared sufficient variance to justify factor extraction. Maximum likelihood (ML) was selected as the method of factor extraction; eigenvalues greater than 1, the Kaiser criterion, and the scree test were checked for agreement. The reliability of the scale was calculated using the Cronbach’s alpha coefficient [40]. Features of concurrent validity were explored by a series of two-tailed Pearson linear correlations. Discriminant Validity was assessed using Analysis of Variance (ANOVA). Statistical analyses were conducted using SPSS 18.0 (SPSS Inc., Chicago, IL, USA).

## 3. Results

### 3.1. Descriptive Statistics

Means and SD of the two samples are shown in Table 1.

### 3.2. Reliability and Construct Validity 

Cronbach’s alpha coefficients for the scales of the SI and for the scales of the Seven Domains Addictions Scale (7DAS) suggested good reliability (see Table 2 and Table 3). Examination of the scree plots [41], and percentages of variance accounted for, revealed the presence of one factor for each scales of SI and for each scale of the Seven Domains Addictions Scale (7DAS). The EFA showed factor structures with one principal dimension for all scales (see Table 2 and Table 3).

### 3.3. Convergent Validity

The ABQ (SI and 7DAS) showed significant correlations with the majority of measures used to assess concurrent validity (see Table 4 and Table 5).

### 3.4. Discriminant Validity

ANOVA results showed that the clinical group obtained significantly higher values for most variables analyzed than the nonclinical group, indicating a good discriminant validity of the ABQ (see Table 6 and Table 7).

## 4. Discussion

The aim of this work was to illustrate the theoretical and clinical model behind the ABQ and to verify its psychometric properties (factor structure, reliability, and validity). The importance of creating this new measure for assessing substance-related and addictive disorders lies in the fact that the ABQ is based on a novel and integrated model of addiction that can provide an empirically grounded framework for understanding, assessing, and treating the dysfunctional processes at the basis of addictive behaviors. In fact, we believe that the seven domains of addiction identified in the ABQ allow clinicians and researchers to identify the dysfunctional processes and dynamics that foster the development and maintenance of addiction. From a behavioral perspective, addictive behaviors are reinforced by both positive (the pleasure linked to the behaviors) and negative stimuli (the painful states involved in withdrawal symptoms), which fosters impulsivity and compulsivity, as well as obsessiveness toward the behavior. However, from a developmental perspective, addictive behaviors may also emerge from a need to regulate painful internal states, and/or to dissociate traumatic memories and/or to deal with attachment insecurities and mistrust in close relationships [2,3,42,43,44]. These considerations were useful in the development of a measure that provides clinicians and researchers with a dimensional picture of the severity of the addictive behaviors and their related maladaptive domains of functioning (Separation anxiety, Affect dysregulation, Somatoform and psychological dissociation, Childhood traumatic experiences, Impulse dyscontrol, Compulsive behavior and ritualization, Obsessive thoughts). In line with this, we created a measure of addiction useful for diagnosis and for the repeated measurement of client status over the course of therapy and at termination. In fact, by evaluating the subjective conditions underlying the specific additive behavior, the ABQ allows the clinician to develop and monitor an individualized therapeutic intervention in favor of the patient. The ABQ can be adapted to different clinical settings; in fact, the theoretical principles behind the ABQ make possible its use in different settings and by clinicians of different orientations. In the present research, the ABQ showed a clear and clinically relevant factor structure, with independent and robust dimensions. Each dimension showed good values of internal consistency. Regarding aspects of concurrent validity, the ABQ showed good correlations with the most common self-reported measure used for the assessment of the different types of addiction.

Finally, the ABQ showed good levels of discriminant validity, and this testifies to the ability of the ABQ scales to distinguish between clinical and normal population. An exception was observed in the SI scale assessing problematic Internet use. This likely results from the current lack of formal diagnosis of Internet addiction or Internet gaming disorder in current diagnostic manuals, which makes it difficult to recruit patients who are seeking treatment for such problems. In fact, very few participants in the clinical group presented concurrent problems with Internet use, and their excessive use of the Internet was mostly related with PG. However, the positive correlation of IAT scores with the scales of Impulse dyscontrol, Compulsive behavior and ritualization, and Obsessive thoughts of the 7DAS suggest that the ABQ may be used for screening problems linked to excessive Internet use, even though the SI scale concerning problematic Internet use may need further empirical examination. Further caution should be needed in the assessment of problematic Internet use, because the current trend in research is to identify specific domains of addiction to Internet applications, such as Internet gaming disorder, even though this trend is criticized among many scholars [45].

As with all research, this study comes with several limitations. First, we did not differentiate participants in the clinical group based on their specific diagnosis. This allowed us to improve the statistical power of our study, but at the cost of obscuring potentially specific differences in symptoms and way of functioning within the clinical group. Second, even though the theoretical rationale behind the ABQ may be considered as comprehensive, and its scales demonstrated to be clinically meaningful, addictive disorders are biopsychosocial disorders and many factors may contribute to their development, including other factors that are not comprised in the ABQ. Finally, this study represents an initial validation of a promising measure, but further validation with clinical and nonclinical samples are needed to better understand the strengths and limitations of the ABQ with respect to other comprehensive measures of addictive behaviors.

These limitations notwithstanding, our findings suggest that the ABQ is a comprehensive and easily administered and scored instrument for assessing addiction in adults, with good psychometric properties. Therefore, it can be used in both research and clinical practice for the assessment of addictive behaviors and for the development of individualized treatment plans directed to people who suffer from substance-related and addictive disorders.

## Figures and Tables

**Table 1:** Demographics variables of the samples.

	Clinical Sample (*N* = 515)		Control Sample (*N* = 183)		Total Sample (*N* = 698)	
Age						
	M = 39.03, SD = 11.64		M = 29.09, SD = 8.62		M = 36.44, SD = 11.77	
Sex						
	*n*	%	*n*	*%*	*n*	*%*
Male	398	77.3	84	45.9	482	69.1
Female	111	21.6	98	53.6	209	29.9
Missing values	6	1.2	1	0.5	7	1.0
Nationality						
Italian	468	90.9	179	97.8	647	92.7
Other	27	5.2	1	0.5	28	4.0
Missing values	20	3.9	3	1.6	23	3.3
Marital status						
Single	284	55.1	127	69.4	411	58.9
Married	117	22.7	45	24.6	162	23.2
Separated	46	8.9	1	0.5	47	6.7
Divorced	26	5.0	4	2.2	30	4.3
Widowed	7	1.4	2	1.1	9	1.3
Cohabitant	30	5.8	3	1.6	33	4.7
Missing values	5	1.0	1	0.5	6	0.9
Professional condition						
Unemployed	237	46.0	22	12.0	259	37.1
Looking for first job	15	2.9	10	5.5	25	3.6
Entrepreneur	19	3.7	16	8.7	35	5.0
Employee	63	12.2	39	21.3	102	14.6
Artisan	31	6.0	4	2.2	35	5.0
Trader	16	3.1	4	2.2	20	2.9
Armed forces	2	0.4	2	1.1	4	0.6
Housewife	6	1.2	5	2.7	11	1.6
Student	18	3.5	69	37.7	87	12.5
Retired	29	5.6	1	0.5	30	4.3
Other	72	14.0	9	4.9	81	11.6
Missing values	7	1.4	2	1.1	9	1.3
Study degree (years of education)						
Elementary school (5 years)	33	6.4	0	0	33	4.7
Middle School diploma (8 years)	250	48.5	22	12.0	272	39.0
High School diploma (13 years)	199	38.6	99	54.1	298	42.7
University degree (16 years)	11	2.1	29	15.8	40	5.7
Master’s degree (18 years)	12	2.3	25	13.7	37	5.3
Post-Lauream Specialization (22 years)	5	1.0	8	4.4	13	1.9
Missing values	5	1.0	0	0	5	0.7

**Table 2:** Factor Analysis and Cronbach’s alpha for the SI.

Scales	*n* Items	Factors	Variance Explained	Alpha	KMO/Bartlett
Substances	8	1	57.33%	89	(KMO) = 0.89; Bartlett <0.001
Alcohol	7	1	57.86%	87	(KMO) = 0.87; Bartlett < 0.001)
Gambling	12	1	58.67%	93	(KMO) = 0.93; Bartlett < 0.001)
Internet	7	1	54.36%	84	(KMO) = 0.86; Bartlett < 0.001)

**Table 3:** Factor Analysis and Cronbach’s alpha for the 7DAS.

Scales	*n* Items	Factors	Variance Explained	Alpha	KMO/Bartlett
Separation anxiety	7	1	49.72%	84	(KMO = 0.85; Bartlett < 0.001)
Affect dysregulation	7	1	45.85%	81	(KMO) = 0.87; Bartlett < 0.001)
Somatoform and psychological dissociation	7	1	42.06%	75	(KMO) = 0.83; Bartlett < 0.001)
Childhood traumatic experiences	7	1	52.13%	85	(KMO) = 0.86; Bartlett < 0.001)
Impulse Dyscontrol	7	1	39.62%	73	(KMO) = 0.83; Bartlett < 0.001)
Compulsive behavior and ritualization	7	1	42.17%	78	(KMO) = 0.85; Bartlett < 0.001
Obsessive thoughts	7	1	54.08%	87	(KMO) = 0.87; Bartlett < 0.001)

**Table 4:** Correlations between Severity Index and the scales administered for convergent validity.

Scales	Substances	Alcohol	Gambling	Internet
TAS-20	0.34 **	0.26 **	0.17 *	0.23 **
BIS-11	0.39 **	0.20 **	0.13	0.06
PTI Sec	−0.15 *	−0.12	−0.08	−0.05
PTI Pre	0.23 **	0.34 **	0.05	0.35 **
PTI Avo	−0.01	0.11	0.01	0.21 **
PTI Unr	0.26 **	0.39 **	0.05	0.31 **
IAT	0.12	0.22 **	0.05	0.63 **
SOGS	0.06	−0.24 **	0.41 **	−0.21 **
YB-O	0.21 **	0.18 **	0.24 **	0.37 **
YB-C	0.01	0.15 *	0.17 *	0.23 **
DES-II	0.36 **	0.41 **	0.10	0.30 **
AUDIT	0.28 **	0.71 **	−0.01	0.35 **
TEC	0.11	0.28 **	−0.13	0.43 **
SDQ-5	0.16 *	0.30 **	0.08	0.23 **

* *p* < 0.01; ** *p* < 0.001. Note. TAS-20 = Twenty-Items Toronto Alexithymia Scale; BIS-11= Barratt Impulsiveness Scale; PTI Sec = Psychological Treatment Inventory-Secure Scale; PTI Pre = Psychological Treatment Inventory-Preoccupied scale; PTI Avo = Psychological Treatment Inventory-Avoidant Scale; PTI Unr = Psychological Treatment Inventory-Unresolved Scale; IAT= Internet Addiction Test; SOGS = South Oaks Gambling Screen; YB O = Yale-Brown Obsessive Compulsive Scale—Obsessive Scale; YBC =Yale-Brown Obsessive Compulsive Scale—Compulsive Scale; DES-II = Dissociative Experience Scale-II; AUDIT = Alcohol Use Disorders Identification Test; TEC= Traumatic Experiences Checklist; SDQ-5 = Somatoform Dissociation Questionnaire-5.

**Table 5:** Correlations between 7DAS and the scales administered for convergent validity.

Scale	Domain 1 Separation Anxiety	Domain 2 Affect Dysregulation	Domain 3 Somatoform and Psychological Dissociation	Domain 4 Childhood Traumatic Experiences	Domain 5 Impulse Dyscontrol	Domain 6 Compulsive Behavior and Ritualization	Domain 7 Obsessive Thoughts
TAS-20	0.46 **	0.59 **	0.49 **	0.12 *	0.42 **	0.35 **	0.50 **
BIS-11	0.31 **	0.45 **	0.42 **	0.07	0.49 **	0.39 **	0.45 **
PTI Sec	−0.29 **	−0.29 **	−0.12 *	−0.13 *	−0.17 **	−0.10 *	−0.21 **
PTI Pre	0.69 **	0.50 **	0.36 **	0.21 **	0.37 **	0.38 **	0.47 **
PTI Avo	0.10 *	0.12 *	0.16 **	0.14 *	0.20 **	0.14 *	0.19 **
PTI Unr	0.35 **	0.31 **	0.35 **	0.18 **	0.31 **	0.32 **	0.35 **
IAT	0.11 *	0.20 **	0.19 **	0.02	0.26 **	0.28 **	0.25 **
SOGS	−0.04	0.05	−0.02	–0.08	−0.03	−0.08	−0.03
YB O	0.23 **	0.29 **	0.30 **	0.04	0.29 **	0.34 **	0.32 **
YB C	0.22 **	0.22 **	0.22 **	0.08	0.25 **	0.32 **	0.26 **
DES-II	0.34 **	0.43 **	0.59 **	0.24 **	0.42 **	0.46 **	0.45 **
AUDIT	0.31 **	0.25 **	0.22 **	0.13 *	0.29 **	0.27 **	0.23 **
TEC	0.24 **	0.20 **	0.22 **	0.38 **	0.31 **	0.19 **	0.23 **
SDQ-5	0.23 **	0.25 **	0.40 **	0.24 **	0.33 **	0.33 **	0.24 **

* *p* < 0.01; ** *p* < 0.001. Note. TAS = Twenty-Items Toronto Alexithymia Scale; BIS= Barratt Impulsiveness Scale; PTI Sec = Psychological Treatment Inventory-Secure Scale; PTI Pre = Psychological Treatment Inventory-Preoccupied scale; PTI Avo = Psychological Treatment Inventory-Scala Avoidant Scale; PTI Unr = Psychological Treatment Inventory-Unresolved Scale; IAT= Internet Addiction Test; SOGS = South Oaks Gambling Screen; YB O = Yale-Brown Obsessive Compulsive Scale—Obsessive Scale; YB C =Yale-Brown Obsessive Compulsive Scale—Compulsive Scale; DES-II = Dissociative Experience Scale Revised; AUDIT = Alcohol Use Disorders Identification Test; TEC= Traumatic Experiences Checklist; SDQ-5 = Somatoform Dissociation Questionnaire-5.

**Table 6:** Discriminant validity of the SI.

	Control Sample		Clinical Sample		df	F	*p*
Severity Index	M	SD	M	SD			
Substances	3.53	3.42	12.73	8.16	1305	32.36	0.001
Alcohol	2.61	2.88	6.15	6.45	1403	29.89	0.001
Gambling	13.28	6.99	19.38	9.99	1204	9.63	0.010
Internet	3.78	4.12	4.12	5.14	1252	0.33	0.567

**Table 7:** Discriminant validity of the 7DAS.

	Control Sample		Clinical Sample		df	F	*p*
Seven Domains Addiction Scale	M	SD	M	SD			
Separation anxiety	7.41	5.66	10.22	6.45	1677	26.57	0.001
Affect dysregulation	8.76	4.98	11.82	5.33	1678	45.39	0.001
Somatoform and psych dissociation	2.59	2.78	4.04	4.09	1678	19.51	0.001
Childhood traumatic experiences	2.47	3.68	5.75	5.97	1674	47.60	0.001
Impulse dyscontrol	7.47	4.39	10.39	5.36	1679	43.16	0.001
Compulsive behavior and ritualization	5.16	4.18	7.95	5.01	1675	45.02	0.001
Obsessive thoughts	9.62	6.15	11.62	5.91	1677	15.06	0.001
